# Job crafting promotes internal recovery state, especially in jobs that demand self-control: a daily diary design

**DOI:** 10.1186/s12889-021-11915-1

**Published:** 2021-10-19

**Authors:** Yanwei Shi, Zhuang She, Dan Li, Hui Zhang, Kuihuan Niu

**Affiliations:** 1 Department of Human Resource Management, Shanghai Normal University, Shanghai, 200234 China; 2 Affiliated Mental Health Center (ECNU), School of Psychology and Cognitive Science, East China Normal University, Shanghai, 200062 China; 3 Hainan Medical University, Haikou, 570216 China; 4 School of Sociology, Huazhong University of Science and Technology, Wuhan, 430000 China; 5 School of Marxism, Shanghai Institute of Technology, Shanghai, 200062 China

**Keywords:** Conservation of resources, Daily diary, ego depletion, Job crafting, Recovery from work, Self-control demands at work

## Abstract

**Background:**

Research on how employees recover from work has focused primarily on recovery during non-work hours (external recovery) rather than recovery during work hours (internal recovery). Using the conservation of resources theory as a conceptual framework, we tested whether job crafting promotes an internal recovery state, and examined the processes that explain this association.

**Methods:**

Using the daily diary method, 120 full-time employees provided information before and after work for 5 days by rating job crafting, ego depletion, self-control demands at work, fatigue and vigor.

**Results:**

The results of multilevel modeling showed that after controlling for employees’ fatigue and vigor before work, daily job crafting predicted significantly better internal recovery (greater vigor and lower fatigue at the end of workday), and this association was mediated by lower ego depletion. The links between job crafting and internal recovery were stronger for employees with high self-control demands at work.

**Conclusions:**

This study extends recovery research by examining internal recovery as well as job crafting as its antecedent. Further, the present study suggests that managers may consider encouraging and offering job crafting interventions for employees to achieve internal recovery state.

## Background

Employees often have heavy workloads and face a lot of job stress. Job stressors draw on the employee’s resources and may lead to strain that can compromise health and performance [1, 2]. Thus, employees need to recover from work during off-job time to stay healthy and maintain well-being [3]. Previous studies have shown that recovery outside of job time promotes employees’ well-being and better functioning at work. For example, recovery is positively related to life satisfaction [4], the next day’s work engagement [5, 6] and task performance [7].

However, previous studies might only tell half of the story. In the literature on recovery, researchers have mainly concentrated on recovery occurring after work in non-work contexts (external recovery) [4–7], even though recovery from work can start during the work day in the work context (internal recovery). This aspect of recovery has received little attention from researchers. One exception is a study [8] that found that internal recovery during the work day increased the probability that employees would start their next working day in an optimally recovered state. Therefore, it is important to fill this research gap in order to facilitate employees’ internal recovery even when facing high work pressures and workloads.

**Fig. 1:**
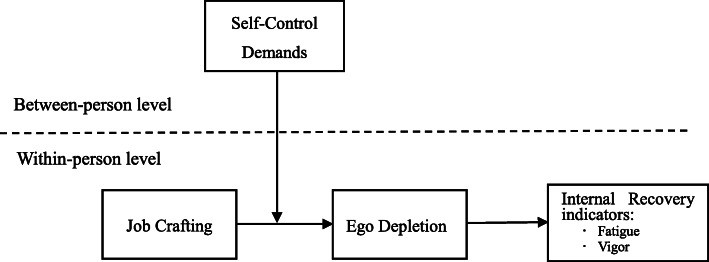
Proposed research model

According to the conservation of resources (COR) theory [9, 10], recovery from work occurs when employees replenish psychological, emotional and physical resources that are depleted during work. This recovery during work prevents further depletion of resources after work. One way that recovery can be accomplished is by employees changing the demands of their job in relation to their job resources, also called job crafting [11, 12]. Job crafting helps the employee gain resources and prevent the loss of resources [11, 12]. In the current study we focus on the possibility that job crafting helps employees to maintain a state of internal recovery. We also explore a potential underlying mechanism (ego depletion) and a boundary condition (self-control demands at work) in the relationship. In addition, consistent with previous studies [8], we focus on fatigue and vigor as indicators of employees’ internal recovery state. Figure 1 summarizes the relationships tested in this study.

The current study makes to two main contributions to the literature. First, to our knowledge, the effect of job crafting on internal recovery state is uncharted territory. Based on COR theory [10], we argue that by job crafting [12], employees can replenish and prevent further depletion of resources that are depleted during the workday. By maintaining adequate resources, these employees are less likely to experience ego depletion [13], which in turn positively impacts internal recovery state [14]. In sum, the current study makes a contribution to understand potential mechanisms in the relationship between job crafting and internal recovery state. Further, this study has implications for how employers and employees can facilitate employees’ recovery after work.

The second contribution has to do with COR theory’s principle of the gain paradox, the idea that resource gains become more important when resource loss circumstances are high [15]. Based on this principle, the positive effect of job crafting on internal recovery becomes more important in an environment with high resource losses, namely a job with high demands for self-control. Jobs that demand a high level of self-control are stressful and entail resource losses that can have detrimental effects on employees. Nevertheless, self-control demands have become an integral part of the job in many occupations [16, 17]. Our study extends the literature by testing whether job crafting has a differential effect on the internal recovery state of employees with high or low self-control demands at work.

## Literature and theoretical background

### Job crafting

Job crafting is an employee-initiated behavior to redesign one’s job from bottom-up. There are two perceptive on job crafting. Wrzesniewski and Dutton [18] proposed that job crafting is the employee-initiated physical and cognitive changes to their jobs to enhance job meaning, which includes changing relational boundaries, task boundaries and cognitive boundaries of the job.

Tims et al. [12] later proposed a more comprehensive concept of job crafting based on the job Demands-Resource Model. According to this model, jobs are characterized by demands and resources. Job demands require employees’ sustained effort and have certain costs. By contrast, job resources reduce the effect of job demands and associated costs, making it easier to achieve work goals and personal development [19]. Thus, in the Demands-Resource Model, job crafting refers to the self-initiated changes that employees make in their own job demands and job resources to attain and/or optimize their personal or work goals.

Tims et al. [12] also proposed that job crafting has four dimensions: (1) increasing structural job resources (i.e., mobilizing job characteristics that help to achieve work goals and develop the self, such as opportunities for development, autonomy, or skill variety), (2) increasing social job resources (i.e., mobilizing job characteristics in the relational sphere, such as seeking social support, supervisory coaching, or performance feedback), (3) increasing challenging job demands (i.e., creating access to job demands that require effort but are rewarding when attained; for example, starting new projects), and (4) decreasing hindering job demands that hinder productivity (i.e., making sure one’s work is less demanding, for example by ensuring the work is emotionally less taxing).

Job crafting can occur as part of an official re-structuring of a job or job responsibilities, but employees also adjust the workplace environment and job design on a daily basis [20]. Previous research showed that daily fluctuations in job crafting ranged between 31% (increased challenges) and 78% (decreased demand) [21]. In an era of increasingly complex and ever-changing work, job crafting is associated with employees’ job satisfaction [22], work engagement [21, 23], job performance [24], and well-being [25, 26]. In addition, job crafting could prevent resource loss by relieving burnout [27], decreasing exhaustion [28], and relieving negative affect [26].

Although previous studies on job crafting found that distinct dimensions of job crafting have different effect on employee [21], different job crafting behaviors are not necessarily mutually exclusive and can be deployed simultaneously [29]. The goal of the present study was to explore the potential effect of job crafting in general, rather than the potential differential effects of specific dimensions of job crafting. Consistent with this goal, we considered job crafting as one factor rather than three factors and used an overall composite score on a measure of job crafting to test our hypotheses. However, we also provide results from supplemental analyses on each subscale of this measure (i.e., measures of specific dimensions of job crafting).

### COR theory

COR theory provides a foundation for understanding how job crafting affects vigor and fatigue. COR theory proposes that individuals seek to prevent resource losses and to gain resources to cope with job demands [9, 10]. This process is especially important for employees who have fewer resources to start with. If these resources are not replenished, these employees are at risk of losing even more resources, resulting in strain and difficulty in attaining an internal recovery state. Resources such as support and energy help employees to better address environmental demands. According to COR theory, employees need to gain resources or replenish consumed resources to recover from work.

Job crafting can potentially increase important resources such as supervisor supports, social relations [12, 20, 30] and reduce resource loss by relieving fatigue and decreasing burnout [30–32]. Thus, according to COR theory and the empirical evidence of the benefits of job crafting, it is reasonable to argue that job crafting increases resources and prevents resource loss, thereby reducing stress and promoting internal recovery from work demands.

The gain paradox principle of COR theory is also helpful in understanding why job crafting is more important in some job contexts than others [15]. According to this principle, employees who encounter high self-control demands at work are more likely to experience resource loss [33] and job crafting becomes more important for them, thereby reducing ego depletion and increasing internal recovery state.

### Hypothesis development

#### Mediating effect of ego depletion

Ego depletion refers to a state in which there has been an exhaustion of resources for changing behaviors or pursuing goals [34, 35]. Based on COR theory [15], individuals who have more resources are less vulnerable to ego depletion. We propose that job crafting provides employees the opportunity to gain resources and reduce the loss of resources, and thus to be lower their risk of ego depletion.

Specifically, job crafting might prevent employee ego depletion in the following ways. First, job crafting could increase job resources, and employees who possess resources are better equipped to handle stressful circumstances and more likely to avoid problematic situations [10]. In line with this proposition, increasing structural job resources and social job resources via job crafting enables employees to handle high job demands, leading to less ego depletion. For example, previous studies demonstrated that job crafting is negatively related to burnout and exhaustion [27, 28].

Second, job crafting could prevent ego depletion by creating more challenging job demands. Challenging job demands require extra resources from employees but do not necessarily cause resource loss, as the positive emotions, self-efficacy and personal growth provided by challenging job demands are important resources for employees [34, 35]. Thus, although challenging job demands do require extra effort they do not have an energy-depleting effect [36]. For example, Crawford et al. [37] found that increasing challenging job demands contributed to lower burnout.

Third, job crafting may help prevent ego depletion by reducing hindering job demands [19]. Decreasing hindering job demands allows employees to focus their efforts on core work tasks and to restore energy [12]. For example, employees may protect themselves from ego depletion via minimizing contact with people whose problems affect them emotionally. Further, decreasing hindering job demands is related to decreased burnout and exhaustion [27, 28]. Taken together, this evidence leads us to we believe that employees who decrease hindering job demands via job crafting are at lower risk of ego depletion.

Thus, based on COR theory and previous research, we expected that employees’ job crafting would predict lower ego depletion on a day-to-day basis at work.

##### Hypothesis 1

Daily job crafting will be negatively related to daily ego depletion at work.

COR theory proposes that individuals who have fewer resources are more vulnerable to further resource loss and less capable of resource gain; in this group, resource loss is also more likely to result in strain [15]. In line with this proposition, employees who experience ego depletion at work have fewer resources to deal with any additional job demands. For example, if employees are in a state of depletion at work, they would find subsequent job tasks more demanding and need more resources to overcome non-task distractions. Further, depleted individuals have fewer resources to replenish the depleted job-related resources and build new resources (e.g., energy, positive mood). As a result of this continued resource depletion and lack of recovery of depleted resources, employees are likely to experience increased fatigue and decreased vigor.

There is some initial evidence that supports the above view. Researchers have demonstrated that self-regulatory resource depletion induced by self-control negatively predicts end-of-day vigor and positively predicts end-of-day fatigue [8]. Similarly, Lanaj et al. [14] found that morning depletion diminished employees’ daily vigor. Thus, there are conceptual and empirical reasons to predict that employees who experience ego depletion will feel more fatigue and less vigor at the end of the workday.

##### Hypothesis 2

Daily ego depletion will be negatively related to end-of-day vigor (2a) and positively related to end-of-day fatigue (2b).

Based on the aforementioned arguments about the relationships between daily job crafting and daily ego depletion, and between daily ego depletion and vigor and fatigue at the end of workday, it might be expected that daily ego depletion would mediate the relationship between job crafting at work and the resources available after work. In terms of COR theory, job crafting is a resource-gaining experience; the gain in resources lowers the risk of ego depletion and allows the employee to deal with additional job demands; and the employee has sufficient resources left at the end of the day.

##### Hypothesis 3

Daily ego depletion will mediate daily job crafting’s association with daily greater vigor (3a) and daily lower fatigue (3b) at the end of workday.

#### Moderating effect of self-control demands at work

To effectively adapt to dynamic and changing working environments, employees cannot rely on rigid, automatic, and habitual behavioral patterns but rather must exert volitional self-control [38]. In addition, different acts of self-control appear to draw on a common regulatory resource, leading to resource loss and ego depletion [39]. Thus, self-control demands at work are an important cause of resource loss for many employees.

Self-control demands at work require employees to exert impulse control, resist distractions and overcome inner resistances [40]. There are three forms of self-control. First, impulse control refers to inhibiting spontaneous, impulsive response tendencies and associated affective states, which manifest in injudicious comments. For example, in the service industry, employees are required to maintain a positive mood at all times when serving customers and not to lose their temper. Another example is some work requires employees to weigh every word before saying something. Second, resisting distractions involves ignoring or resisting distractions evoked by task-irrelevant stimuli. For example, employees must not let themself be distracted by non-work related things to achieve their performance goals. Third, overcoming inner resistances relates to overcoming motivational deficits that result from unappealing tasks [41]. For example, employees need to force themself to get some of work tasks done. Researchers have found that self-control demands at work is associated with work-related outcomes such as increased job burnout [39], decreased subjective vitality [42] and work engagement [43].

The gain paradox principle of COR theory proposes that resource gains and replenishment are especially important for individuals who have not recovered from earlier lost resources [15]. Because employees with high self-control demands at work experience a depletion of limited self-control resources, and are more likely to experience burnout and depression [34, 35], they are likely to be in an ongoing state of resource loss [33]. Compared to employees in jobs with low demands for self-control, those in jobs with high demands for self-control are likely to benefit most from job crafting as a way to reduce ego depletion. Based on these the gain paradox principle, we proposed the following moderation hypothesis:

##### Hypothesis 4

Self-control demands at work will moderate the negative relationship between daily job crafting and daily ego depletion, with the relationship being stronger when self-control demands at work are high.

Combining our previous two hypotheses on mediation and moderation, we also proposed the following moderated mediation hypothesis:

##### Hypothesis 5

Self-control demands at work will moderate the indirect effect of daily job crafting on daily greater vigor (5a) and daily lower fatigue (5b) at the end of workday through daily ego depletion, with the indirect effect being stronger when self-control demands at work are high.

## Method

### Participants and procedure

We recruited 170 employees from various organizations in China to participate in a 5-day diary study. Of the 170 employees who agreed to participate, 151 (88.82%) actually took part. Of these 151 participants, 31 were dropped from the sample because they completed the daily questionnaire on fewer than 3 days. In the final sample of 120 employees, the average age was 29.19 years old (*SD* = 5.63) and the average years of tenure at the current job was 6.13 years (*SD* = 6.99). Forty nine participants were male, and seventy one participants were female. Most held a college degree (57.5%) or a graduate degree (36.7%), and a small number held a high school degree (5.8%).

Participants were approached through the social networks of research assistants involved in this study. After agreeing to take part, they received information about the study procedure and a link to a web-based general questionnaire that asked about demographics and self-control demands at work. The next week, they started to complete daily questionnaires twice a day for a period of 5 consecutive workdays. Each day for 5 days, research assistants sent a link to a web-based questionnaire through WeChat about 1 h before the requested completion time (when getting up in the morning before work and at the end of the workday). The morning questionnaire assessed fatigue and vigor before work. The evening questionnaire assessed fatigue and vigor after work, daily job crafting, and daily ego depletion.

Each participant received 10 Chinese Yuan (approximately 1.4 US dollars) after completing the questionnaire every day. Participants provide informed consent. The present study received the university’s research ethics committee’s approval and the anonymous of participants’ responses was guaranteed.

### Measures

We used the translation and back-translation method to translate the scales that were originally in English into Chinese. Specifically, scales in English (job crafting, ego depletion, fatigue, vigor and self-control demands at work) were first translated into Chinese by a Ph.D. student who was fluent in both English and Chinese. To check the accuracy of the translation, the Chinese versions of the scales were then back-translated into English by another Ph.D. student who was fluent in Chinese and English. Any discrepancies were resolved through discussions among the lead investigator and the two translators.

#### Daily job crafting

Daily job crafting was assessed using the 10-item Job Crafting Questionnaire developed by Petrou and colleagues [20]. The measure includes three subscales: seeking resources (4 items, e.g., “Today, I asked colleagues for advice”), seeking challenges (3 items, e.g., “Today, I asked for more tasks if I finished my work”), and reducing hindering demands (3 items, e.g., “Today, I made sure that my work was mentally less intense”). Each item is rated on a 5-point Likert scale (1 = strongly disagree, 5 = strongly agree), with high scores indicating high job crafting behavior. In the current study, the Cronbach’s alpha of the full scale was 0.83.

#### Daily ego depletion

To measure daily ego depletion, we used the five-item Daily Ego Depletion Scale developed by Bertrams et al. [44], based on the definition of ego depletion proposed by Muraven and Baumeister [35]. One sample item is “At the moment, I feel like my willpower is gone.” Each item is rated on a 4-point Likert scale (1 = not at all, 4 = a great deal). The ratings were averaged across the five items, with higher scores indicating higher ego depletion. In the current study, the Cronbach’s alpha of the scale was 0.90.

#### Daily recovery indicators

Fatigue and vigor (assessed before and after work) were each measured with five items derived from the Mood State Scale [45]. Each item is rated on a 5-point scale (1 = not at all, 5 = very much). Example items are “Now, I feel fatigued” and “Now, I feel vigorous.” High scores mean “worse” for fatigue and “better” for vigor. Using the end-of-workday assessments, the mean Cronbach’s alpha across the work week was 0.71 for fatigue and 0.95 for vigor.

#### Self-control demands at work

Self-control demands at work were assessed using the 15-item Self-Control Demands at Work Scale, which includes three subscales [41]. The three subscales are impulse control (6 items, e.g., “My job requires me never to lose my temper”), resisting distractions (4 items, e.g., “In order to achieve my performance goals, I must not let myself be distracted”), and overcoming inner resistances (5 items, e.g., “Starting on certain tasks sometimes requires that I use a lot of willpower”). Each item is rated on a 5-point Likert scale (1 = not at all, 5 = a great deal). The ratings were averaged across the 15 items, with higher scores indicating higher self-control demands at work. In the current study, the Cronbach’s alpha of the scale was 0.86.

#### Control variables

A previous study demonstrated gender and age differences in indicators of recovery [46]. Therefore, we used gender (male = 1; female = 0) and age (in years) as control variables in the statistical analyses. In addition, we controlled for the daily morning ratings of fatigue and vigor, because morning fatigue and vigor affect individuals’ fatigue and vigor at the end of the workday [8].

### Statistical analysis

Given that daily repeated measurements of job crafting, ego depletion, and recovery indicators of fatigue and vigor (Level 1) were nested within individuals (Level 2), we used multilevel analysis to test our hypotheses with Mplus Version 7.02 [47]. We tested all our hypotheses in a comprehensive moderated mediation model. Specifically, we specified random slopes for the Level 1 effect of daily job crafting on ego-depletion, as well as the effects of ego-depletion on end-of-workday fatigue and vigor. We also specified random intercepts at Level 2 for ego depletion, fatigue, and vigor, and allowed the random slopes to covary with the random intercepts (e.g., the random slope for the effect of daily job crafting on ego-depletion was allowed to covary with the random intercept of ego depletion). To test the cross-level moderation, we used self-control demands at work to predict the random slope for the effect of daily job crafting on ego-depletion and the random intercept of ego depletion at Level 2.

To facilitate the interpretation of the findings, all Level-1 predictors (e.g., job crafting) were person-mean centered to obtain unbiased estimates of the relationships at the intra-individual level [48]. The Level-2 variable, self-control demands at work, was grand-mean centered. Daily fatigue and vigor in the morning (Level 1), and gender and age (Level 2) were entered as control variables.

## Results

### Preliminary analyses

First, we conducted multilevel confirmatory factor analyses via Mplus [49]. The fit indices of the five–factor model were satisfactory: χ2/*df* = 2.27, CFI = 0.92, TLI = 0.90, RMSEA = 0.06, SRMR = 0.07 at the within-person level and 0.09 at the between-person level.

Then, we calculated the means, standard deviations, and correlations among all variables. Results showed that job crafting was positively related to vigor at the end of the workday (*r* = 0.34, *p* < 0.001), and negatively related to fatigue at the end of the workday (*r* = − 0.17, *p* < 0.05) and to ego depletion (*r* = − 0.21, *p* < 0.01). Additionally, ego depletion was negatively related to vigor at the end of the workday (*r* = − 0.27, *p* < 0.001), and positively related to fatigue at the end of the workday (*r* = 0.75, *p* < 0.001). These results provided preliminary support for our Hypotheses.

Finally, to examine whether variables in the study varied within individuals, we tested a null model that included only the intercept to calculate intraclass correlations (ICC) for each variable. Table 1 shows the amount of variance of each study variable that was explained by within-person variance. The amount of within-person variance was 60% for job crafting, 68% for ego depletion, 63% for end-of-workday fatigue, and 83% for end-of-workday vigor. Therefore, it was appropriate to use a multilevel approach to test our hypotheses.

**Table 1:** Means, Standard Deviations, and Correlations among Study Variables

Variable	*M*	*SD*	ICC	1	2	3	4	5	6	7	8	9
**Between-person level**												
1.Gender	0.61	0.49	–	–								
2.Age	29.19	5.63	–	0.05	–							
3.Self-control demands	3.56	0.55	–	0.03	−0.16	–						
**Within-person level**												
4. Job crafting	3.46	0.68	0.40	0.10	−0.19	0.34^**^	–					
5. Ego depletion	2.43	0.92	0.32	0.15	−0.04	0.47^**^	−0.21^**^	–				
6. Fatigue_(end of work-day)_	2.57	1.06	0.37	0.11	0.16	0.14	−0.17^*^	0.75^***^	–			
7. Vigor_(end of work-day)_	3.48	0.64	0.17	−0.35^*^	0.17	0.03	0.34^***^	−0.27^***^	−0.36^***^	–		
8. Fatigue_(morning)_	2.34	1.02	–	−0.56	0.32	−0.13	0.05	−0.04	−0.06	0.06	–	
9. Vigor_(morning)_	3.10	0.90	–	0.23	0.12	−0.33	−0.04	0.01	−0.01	− 0.04	−0.50^***^	–

*Note. N* = 120 (between-person level); 528 (within-person level). ^*^p < 0.05, ^**^p < 0.01, ^***^p < 0.001

### Hypothesis testing

#### Direct and mediated effects

Table 2 shows that after the control variables were entered, daily job crafting positively predicted daily ego depletion (B = − 0.25, *SE* = 0.11, *p* < 0.05). Daily ego depletion positively predicted fatigue at the end of workday (B = 0.71, *SE* = 0.05, *p* < 0.001) and negatively predicted vigor at the end of workday (B = − 0.15, *SE* = 0.05, *p* < 0.01). These results supported Hypotheses 1 and 2.

**Table 2:** The indirect effect of daily ego depletion on the relationship between job crafting and fatigue and vigor at the end of the workday

Variable	Ego depletion		Fatigue_(at the end-of-workday)_		Vigor_(at the end-of-workday)_	
	B	*SE*	B	*SE*	B	*SE*
**Within-person level**						
Job crafting	−0.25^*^	0.11	−0.02	0.05	0.33^***^	0.05
Ego depletion			0.71^***^	0.05	−0.15^**^	0.05
Fatigue_(morning)_	−0.05	0.04	−0.05	0.04	0.01	0.03
Vigor_(morning)_	−0.03	0.05	−0.02	0.04	−0.01	0.03
Residual variance at within Level	0.55^***^	0.06	0.41^***^	0.04	0.30^***^	0.03
**Between-person level**						
Gender	0.06	0.11	0.15	0.14	−0.13^*^	0.06
Age	0.01	0.02	0.01	0.02	0.01	0.01
Residual variance at between Level	0.26^***^	0.07	0.41^***^	0.06	0.03	0.02

*Note. N* = 120 (between-person level); 528 (within-person level); ^*^p < 0.05, ^**^p < 0.01, ^***^p < 0.001

As shown in Table 3, the indirect effect of daily job crafting on fatigue at the end of workday via daily ego depletion was significant (indirect effect = − 0.18, 95% *CI* [0.01, 0.07]). The indirect effect of daily job crafting on vigor at the end of workday via daily ego depletion was significant (indirect effect = 0.05, 95% *CI* [0.01, 0.07]). These results supported Hypotheses 3.

**Table 3:** The indirect effects of job crafting on internal recovery state

Mediator	Dependent variables	Indirect Effect	95%CI
Ego depletion	fatigue	−0.18	[0.01, 0.07]
	vigor	0.05	[0.01, 0.07]

#### Cross-level moderated mediation effects

**Fig. 2:**
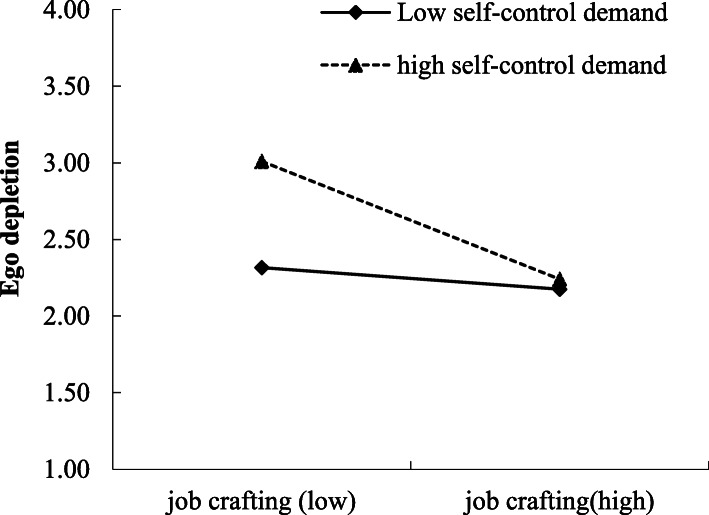
Cross-level moderating effect of self-control demand on the relation between job crafting and ego depletion

We tested a cross-level moderation effect of self-control demands at work on the within-person relationship between job crafting and ego depletion (Hypothesis 4). As shown in Table 4, after entering the control variables, the interaction between job crafting and self-control demands at work negatively predicted ego depletion (B = − 0.23, *SE* = 0.12, *p* < 0.05). Simple slopes analyses showed that the effect of job crafting on ego depletion was stronger for employees with high self-control demands at work (B_*simple*_ = − 0.46, *p* < 0.01) than those with low self-control demands at work (B_*simple*_ = − 0.25, *p* < 0.05; see Fig. 2). Therefore, Hypothesis 4 was supported.

**Table 4:** Regression results for moderated meditation effect

Variable	Ego depletion		Fatigue_(at the end-of-workday)_		Vigor_(at the end-of-workday)_	
	B	*SE*	B	*SE*	B	*SE*
***Within-person level***						
Job crafting	−0.33^**^	0.10	−0.03	0.05	0.28^***^	0.06
Ego depletion			0.71^***^	0.05	−0.15^**^	0.05
Fatigue_(morning)_	−0.04	0.04	−0.05	0.05	0.01	0.03
Vigor_(morning)_	−0.02	0.05	−0.02	0.04	−0.01	0.03
Residual variance at within Level	0.48^***^	0.05	0.41^***^	0.04	0.30^***^	0.03
***Between-person level***						
Gender	0.09	0.12	0.16	0.14	−0.14	0.07
Age	0.01	0.01	0.01	0.01	0.00	0.01
Self-control demands	0.15^*^	0.05				
Residual variance at between Level	0.28^***^	0.06	0.42^***^	0.06	0.07^**^	0.02
**Cross-level interaction**						
Job crafting × Self-control demands	−0.23^*^	0.12				
Residual variance at cross-level	0.27^*^	0.15				

*Note. N* = 120 (between-person level); 528 (within-person level); ^*^p < 0.05, ^**^p < 0.01, ^***^p < 0.001

To test Hypotheses 5a and 5b, we calculated the differences between the indirect effects at high and low levels of self-control demands for each outcome variable. As shown in Table 5, the indirect effect of job crafting on vigor when self-control demands at work were high (B = 0.35, 95% *CI* [0.13, 0.56], *p* < 0.05) was significantly stronger than when self-control demands at work were low (B = 0.07, 95% *CI* [0.02, 0.12], *p* < 0.05), with a difference of 0.28 and a 95% *CI* [0.03, 0.53]. Thus, Hypothesis 5a was supported. Further, as shown in Table 6, the indirect effect of job crafting on fatigue when self-control demands at work were high (− 0.33, 95% *CI* [− 0.47, − 0.18], *p* < 0.05) was not significantly different from when self-control demands at work were low (− 0.24, 95% *CI* [− 0.40, − 0.08], *p* < 0.001), with a difference of − 0.09 and a 95% CI [− 0.23, 0.06]. Therefore, Hypothesis 5b was not supported.

**Table 5:** Conditional indirect effects of job crafting on vigor at different values of self-control demands

Mediator	Self-control demands	Effect	95%CI
Ego depletion	M + 1SD	0.35	[0.13, 0.56]
	M − 1SD	0.07	[0.02, 0.12]

**Table 6:** Conditional indirect effects of job crafting on fatigue at different values of self-control demands

Mediator	Self-control demands	Effect	95%CI
Ego depletion	M + 1SD	−0.33	[−0.47, − 0.18]
	M − 1SD	−0.24	[− 0.40, − 0.08]

#### Additional analyses

We conducted additional analyses to test the indirect effects of the three dimensions of job crafting on internal recovery state (vigor and fatigue at the end of workday) via ego depletion. Results showed that ego depletion mediated the relationships between each of the three dimensions of job crafting and internal recovery state. Specifically, the indirect effects of seeking resources, seeking challenges and reducing hindering demands on vigor at the end of workday through ego depletion were 0.03, 95% *CI* [0.01, 0.07]; 0.01, 95% *CI* [0.00, 0.02]; and 0.01, 95% *CI* [0.00, 0.03], respectively.

The indirect effects of seeking resources, seeking challenges and reducing hindering demands on fatigue at the end of workday through ego depletion were − 0.06, 95% CI [0.03, 0.09]; − 0.08, 95% CI [− 0.15, − 0.01]; − 0.12, 95% CI [− 0.22, − 0.02], respectively. These results suggest that the effects of the three dimensions of job crafting were all consistent with the consequences of job crafting in general.

## Discussion

Drawing on COR theory [9, 10, 15], the present study examined the mediating effect of daily ego depletion in the relationship between daily job crafting and internal recovery state (vigor and low fatigue at the end of day), and the moderating effect of self-control demands at work, using a daily diary method. Our results showed that daily ego depletion mediated the effect of daily job crafting on employee vigor and fatigue at the end of day. Moreover, self-control demands at work affected these indirect effects. Specifically, the indirect effect was stronger for employees with high self-control demands at work than employees with low self-control demands at work. This study demonstrates that positive work behavior (job crafting) is an important means of attaining internal recovery from work.

### Contributions to the literature

Our findings contribute to research on recovery from work and to research on job crafting in several ways. First, we extend research on recovery from work by testing how to promote internal recovery. This issue is important because employees spend a third to a half of their day at the workplace and they have little time to participate in recovery activities during non-work time. Furthermore, organizations have a greater opportunity to influence employees’ internal recovery than external recovery [49].

However, most research has focused on how to promote external recovery [50], paying little attention to the question of how to enhance employees’ internal recovery state. The small number of studies on promoting internal recovery have found that it is associated with work pleasure [51, 52] and work engagement [6]. Our study contributes to this small body of research by examining the positive effect of daily job crafting on employees’ daily internal recovery state. The findings showed that daily job crafting positively predicts employee end-of-day vigor, and negatively predicts employee end-of-day fatigue. Thus, we are among the first researchers to address work-related factors affecting employees’ internal recovery state, instead of off-job factors influencing external recovery. Our findings highlight the value of COR theory as a conceptual framework for studying the benefits of job crafting for daily internal recovery.

Second, our study provides support for the resource perspective on how job crafting might promote employees’ internal recovery state. In line with COR, our findings suggest that daily job crafting can reduce resource depletion by increasing social and structural resources and by decreasing hindering job demands. With lower ego depletion, employees will go on to possess even more resources and lose fewer resources [9]; thus, they will have more resources at the end of the workday and experience more vigor and less fatigue. Our findings extend prior work by using a resource perspective to conceptualize the relationship between job crafting and internal recovery from work.

The finding that job crafting negatively predicts employees’ ego depletion is consistent with previous findings showing that job resources and job demands are related to employees’ ego depletion [53]. Further, our finding suggests that the changes that employees make to balance their job demands and job resources with their personal abilities and needs (job crafting) can relieve employees’ ego depletion. This study thus complements and expands the prior literature on the effect of resource loss on recovery [54], by examining the relationship between ego depletion and internal recovery state. The results showed that daily ego depletion is negatively related to end-of-day vigor and positively related to end-of-day fatigue.

Third, the present study contributes to our understanding of whether job crafting is more beneficial in some circumstances than others. Our study was the first to test the moderator of self-control demands at work in the relationship between job crafting and internal recovery state. We found that the circumstance of high demands for self-control at work increased the association between job crafting and recovery. That is, self-control demands moderated this association. Previous studies mainly focused on the moderating role of leadership styles (i.e., servant leadership) [55], personality traits (i.e., optimism) [56] and job resources (i.e., perceived organizational support) [57] in the relationship between job crafting and work-related outcomes. The present study extends the line by testing the boundary role of job demands (self-control demands).

Individuals are inclined to preserve their daily resource levels in order to prevent complete loss of resources [58]. Job crafting could help employees achieve this by increasing job resources and decreasing job demands. As predicted, employees with high self-control demands are motivated to search for job resources and to decrease job demands (that is, to do job crafting). Consistent with the gain paradox principle of COR theory [15], this finding suggests that employees with high self-control demands have fewer resources and are more likely to benefit from job crafting as a way to reduce the risk of ego depletion. For this group, job crafting could be especially helpful in preventing fatigue and promoting vigor at the end of work.

Finally, the present study contributes to a broader understanding of the effects of job crafting. Studies on job crafting have primarily focused on its effect on employees’ work-related outcomes, such as increasing job satisfaction [59], enhancing job performance [24], and reducing turnover intention [60]. However, the potential positive effects of job crafting on employee non-work-related outcomes have been largely overlooked. Our study found that people who are able to craft their jobs have a higher recovery state at the end of the workday, helping us gain understanding on the effects of job crafting on employees.

One strength of the present study is the use of a within-person daily diary method. This method can capture dynamic and short-term relationships between job crafting and its outcomes. The daily diary method has been used to document the positive effects of daily job crafting on work-related outcomes such as work engagement [21], job performance [61]. Our study found that daily job crafting was negatively related to daily ego depletion and end-of-day fatigue, and positively related to end-of-day vigor. These results provide a more holistic picture of how job crafting might be related to an internal recovery state within a short-term time period, even on a day-to-day basis. Given that employees’ tasks or goals may vary from day to day, the daily diary method is useful in uncovering dynamic adjustments in the actions employees take to recover from work-related stressors.

### Practical implications

The results of the current study have several potential practical implications. First, the findings showed how job crafting may offer a new avenue for employees to achieve an internal recovery state. Hence, managers are advised to offer their employees sufficient leeway to determine what tasks are done and how tasks are done. When employees can determine themselves how they do their work, they can choose to modify their work (e.g., increasing job resources or decreasing hindrances) so they can experience more vigor and less fatigue. Previous studies suggested that job crafting interventions are effective in helping employees to adapt their job demands and job resources [62–64], and they can enhance work engagement [64], improve job performance [65] and increase job resources [37]. Thus, we suggest that managers consider encouraging and offering job crafting interventions for employees. Specific intervention steps are described by Van den Heuvel et al. [30]. These include exercises and goal setting aimed at increasing social job resources, increasing challenging job demands, increasing structural job resources, and decreasing hindering job demands. In training, employees may develop personalized job crafting plans, in which they formulate specific job crafting goals. The findings of our study indicate that these goals may refer to small steps an employee can take to change elements in the work content and context.

Because job crafting is a bottom-up job redesign, organizations need to provide support for employees to craft their jobs. A combination of top-down and bottom-up job redesign seems most likely to yield favorable results for employees and organizations at large. Further, to build resource flexibility for job crafting, organizations should focus on hiring employees who are flexible in their skills and behavior, and who can adapt to new roles or new aspects of their job.

Second, our results showed that ego depletion mediates the relationship between job crafting and internal recovery state. Therefore, in addition to providing job crafting interventions, organizations may provide additional resources to help employees decrease ego depletion, which in turn would boost their internal recovery. One way that organizations could decrease employees’ ego depletion would be by meeting employees’ basic psychological needs at work. For example, given that mindfulness is positively related to basic psychological needs [66], managers may provide mindfulness interventions to meet employees’ psychological needs. In addition, interventions that can target ego depletion (e.g., cognitive-behavioral training) can also be taken into consideration to decrease the risk of ego depletion [67]. Managers can implement these intervention programs in the workplace.

Finally, the present diary study confirms that job crafting’s effects of decreasing ego depletion and increasing recovery from work appear to be most beneficial for employees with high self-control demands at work. However, individuals with high self-control demands are likely to ruminate about their work and have difficulty mentally detaching from work [68]. Thus, it is difficult for employees with high self-control demands to effectively engage in recovery activities such as relaxation. Job crafting may be especially useful for these employees. By contrast, employees with low self-control demands have sufficient resources and need less internal recovery [39]. This group might benefit more from other avenues of attaining recovery from job demands. For example, they could take micro-breaks at work or a longer lunch break [69, 70].

### Limitations and future research

There are several limitations of the present study that need to be addressed in future research. First, the data were all collected with self-report questionnaires, which may raise concerns about common method variance. However, in our study, some potential person-level sources of common method variance (e.g., self-control demands at work) were controlled using a within-person design. In addition, most of the variables of interest in the present study (e.g., ego depletion, vigor, fatigue) concern individuals’ own feelings, making them difficult to measure without self-report. Nevertheless, it will be beneficial in future studies to collect data from different sources to replicate our findings. For example, employees’ supervisors or colleagues might be able to report employees’ job crafting behavior.

Second, the current study focused on the relationship between job crafting and internal recovery, but did not control for the effects of internal recovery activities (e.g., micro-breaks at work, restive lunch breaks, etc.). Previous studies have suggested that employees who self-initiate micro-breaks at work experience higher vigor and lower fatigue [71, 72]. Therefore, future research should control for these self-help activities in order to examine the unique effect of job crafting on internal recovery.

Finally, we tested only employees’ self-control demands at work as a moderator of the relationship between job crafting and internal recovery. Previous studies have demonstrated that perceived organizational support also has an important effect on the relationship between job crafting and employees’ attitudes and behavior [57]. Future research could examine perceived organizational support as a context that could reinforce the association between job crafting and internal recovery.

Finally, consistent with other research [29], we used an overall composite score to represent job crafting. Although research has suggested that there are different dimensions of job crafting [21], supplementary analysis showed a similar pattern of results regardless of the type of job crafting that was being assessed. Nevertheless, future researchers could develop hypotheses about these specific domains of job crafting in relation to employee internal recovery.

## Conclusions

In the present study, we examined the day-to-day relationship between job crafting and employee internal recovery state, as well as the mediating role of ego depletion and the moderating role of self-control demands in this relationship. We found that job crafting predicts lower fatigue and greater vigor at the end of workday by preventing ego depletion. This mediation relationship was stronger for employees with high self-control demands at work. The results were consistent with what would be predicted by the COR theory. The current study is an important step forward in examining how positive work behavior such as job crafting affects employees’ internal recovery from work. Moreover, this study provides insights into how employees with high self-control demands achieve internal recovery during the work day.

## Data Availability

Data are available from the first author on reasonable request (Yanwei Shi, Email: syw@shnu.edu.cn).

## References

[1] Sidhu AK, Singh H, Virdi SS, Kumar R (2020). Job stress and its impact on health of employees: a study among officers and supervisors. J Manag Dev.

[2] Vijayan M (2017). Impact of job stress on employees job performance in aavin, Coimbatore. J Organ Human Behav.

[3] Zijlstra FRH, Cropley M, Rydstedt LW (2014). From recovery to regulation: an attempt to reconceptualize ‘recovery from work’. Stress Health.

[4] Park Y, Fritz C (2015). Spousal recovery support, recovery experiences, and life satisfaction crossover among dual-earner couples. J Appl Psychol.

[5] Sonnentag S (2003). Recovery, work engagement, and proactive behavior: a new look at the interface between nonwork and work. J Appl Psychol.

[6] Sonnentag S, Mojza EJ, Demerouti E, Bakker AB (2012). Reciprocal relations between recovery and work engagement: the moderating role of job stressors. J Appl Psychol.

[7] Binnewies C, Sonnentag S, Mojza EJ (2010). Recovery during the weekend and fluctuations in weekly job performance: a week-level longitudinal study examining intra-individual relationships. J Occup Organ Psychol.

[8] Van Hooff ML, Geurts SA (2015). Need satisfaction and employees’ recovery state at work: a daily diary study. J Occup Health Psychol.

[9] Hobfoll SE (1989). Conservation of resources. A new attempt at conceptualizing stress. Am Psychol.

[10] Hobfoll SE (2002). Social and psychological resources and adaptation. Rev Gen Psychol.

[11] Tims M, Bakker AB (2010). Job crafting: towards a new model of individual job redesign. J Industrial Psychol.

[12] Tims M, Bakker AB, Derks D (2012). Development and validation of the job crafting scale. J Vocat Behav.

[13] Baumeister RF, Vohs KD (2007). Self-regulation, ego depletion, and motivation. Social and personality psychology. Compass..

[14] Lanaj K, Johnson RE, Barnes CM (2014). Beginning the workday yet already depleted? Consequences of late-night smartphone use and sleep. Organ Behav Hum Decis Process.

[15] Hobfoll SE, Halbesleben J, Neveu JP, Westman M (2018). Conservation of resources in the organizational context: the reality of resources and their consequences. Ann Rev Organ Psychol Organ Behav.

[16] Cascio W, Borman C, Ilgen DR, Klimoski RJ (2003). Changes in workers, work, and organizations. Handbook of psychology. Vol. 16: industrial and organizational psychology.

[17] Rivkin W, Diestel S, Schmidt KH (2015). Affective commitment as a moderator of the adverse relationships between day-specific self-control demands and psychological well-being. J Vocat Behav.

[18] Wrzesniewski A, Dutton JE (2001). Crafting a job: Revisioning employees as active crafters of their work. Acad Manag Rev.

[19] Demerouti E, Bakker AB, Nachreiner F, Schaufeli WB (2001). The job demands-resources model of burnout. J Appl Psychol.

[20] Petrou P, Demerouti E, Peeters MC, Schaufeli WB, Hetland J (2012). Crafting a job on a daily basis: contextual correlates and the link to work engagement. J Organ Behav.

[21] Bakker AB, Oerlemans WG (2019). Daily job crafting and momentary work engagement: a self-determination and self-regulation perspective. J Vocat Behav.

[22] Bakker AB, Demerouti E, Cooper CL (2014). Job demands-resources theory. Wellbeing.

[23] Kim H, Im J, Qu H (2018). Exploring antecedents and consequences of job crafting. Int J Hosp Manag.

[24] Tims M, Bakker AB, Derks D (2015). Job crafting and job performance: a longitudinal study. Eur J Work Organ Psychol.

[25] Alonso C, Fernández-Salinero S, Topa G (2019). The impact of both individual and collaborative job crafting on Spanish teachers’ well-being. Educ Sci.

[26] Slemp GR, Kern ML, Vella-Brodrick DA (2015). Workplace well-being: the role of job crafting and autonomy support. Psychol Well-Being.

[27] Tims M, Bakker AB, Derks D (2013). The impact of job crafting on job demands, job resources, and well-being. J Occup Health Psychol.

[28] Petrou P, Demerouti E, Schaufeli WB (2015). Job crafting in changing organizations: antecedents and implications for exhaustion and performance. J Occup Health Psychol.

[29] Dierdorff EC, Jensen JM (2018). Crafting in context: exploring when job crafting is dysfunctional for performance effectiveness. J Appl Psychol.

[30] Van den Heuvel M, Demerouti E, Peeters MC (2015). The job crafting intervention: effects on job resources, self-efficacy, and affective well-being. J Occup Organ Psychol.

[31] Petrou P (2013). Crafting the change: the role of job crafting and regulatory focus in adaptation to organizational change.

[32] Lazazzara A, Tims M, de Gennaro D (2020). The process of reinventing a job: a meta–ssynthesis of qualitative job crafting research. J Vocat Behav.

[33] Muraven M, Tice DM, Baumeister RF (1998). Self-control as a limited resource: regulatory depletion patterns. J Pers Soc Psychol.

[34] Baumeister RF, Bratslavsky E, Muraven M, Tice DM (1998). Ego depletion: is the active self a limited resource?. J Pers Soc Psychol.

[35] Muraven M, Baumeister RF (2000). Self-regulation and depletion of limited resources: does self-control resemble a muscle?. Psychol Bull.

[36] Van den Broeck A, De Cuyper N, De Witte H, Vansteenkiste M (2010). Not all job demands are equal: differentiating job hindrances and job challenges in the job demands-resources model. Eur J Work Organ Psychol.

[37] Crawford ER, LePine JA, Rich BL (2010). Linking job demands and resources to employee engagement and burnout: a theoretical extension and meta-analytic test. J Appl Psychol.

[38] Schmidt KH, Diestel S (2012). The relation of self-control demands to job strain: the moderating role of organisational commitment. Appl Psychol.

[39] Schmidt KH, Diestel S (2015). Self-control demands: From basic research to job-related applications. J Person Psychol.

[40] Schmidt KH, Neubach B (2007). Self-control demands: a source of stress at work. Int J Stress Manag.

[41] Neubach B, Schmidt KH (2007). Entwicklung und Validierung von Skalen zur Erfassung verschiedener Selbstkontrollanforderungen bei der Arbeit [Development and validation of scales for measuring different self-control demands at work at work]. Zeitschrift fu¨r Arbeitswis- senschaft.

[42] Gombert L, Rivkin W, Schmidt KH (2020). Indirect effects of daily self-control demands on subjective vitality via ego depletion: how daily psychological detachment pays off. Appl Psychol.

[43] Rivkin W, Diestel S, Schmidt KH (2018). Which daily experiences can foster well-being at work? A diary study on the interplay between flow experiences, affective commitment, and self-control demands. J Occup Health Psychol.

[44] Bertrams A, Unger A, Dickhäuser O (2011). Momentan verfügbare Selbstkontrollkraft — Vorstellung eines Messinstruments und erste Befunde aus pädagogisch-psychologischen Kontexten [momentary available self-control capacity — introduction of a measure and first results from education-psychological contexts]. Zeitschrift für Pädagogische Psychologie.

[45] McNair DM, Lorr M, Droppelman LF (1971). Manual for the profile of mood states.

[46] Purvanova RK, Muros JP (2010). Gender differences in burnout: a meta-analysis. J Vocat Behav.

[47] Muthén LK, Muthén BO (2010). Mplus user’s guide.

[48] Enders CK, Tofighi D (2007). Centering predictor variables in cross-sectional multilevel models: a new look at an old issue. Psychol Methods.

[49] Sianoja M, Kinnunen U, Bloom JD, Korpela K, Geurts SAE (2016). Recovery during lunch breaks: testing long-term relations with energy levels at work. Scand J Work Organ Psychol.

[50] Sonnentag S, Fritz C (2015). Recovery from job stress: the stressor-detachment model as an integrative framework. J Organ Behav.

[51] Demerouti E, Bakker A, Sonnentag S, Fullagar C (2012). Work-related flow and energy at work and at home: a study on the role of daily recovery. J Organ Behav.

[52] Van Hooff ML, Geurts SA, Beckers DG, Kompier MA (2011). Daily recovery from work: the role of activities, effort and pleasure. Work Stress.

[53] Prem R, Kubicek B, Diestel S, Korunka C (2016). Regulatory job stressors and their within-person relationships with ego depletion: the roles of state anxiety, self-control effort, and job autonomy. J Vocat Behav.

[54] Oerlemans WG, Bakker AB (2014). Burnout and daily recovery: a day reconstruction study. J Occup Health Psychol.

[55] Harju LK, Schaufeli WB, Hakanen JJ (2018). A multilevel study on servant leadership, job boredom and job crafting. J Manag Psychol.

[56] Thun S, Bakker AB (2018). Empowering leadership and job crafting: the role of employee optimism. Stress Health.

[57] Cheng JC, Chen CY, Teng HY, Yen CH (2016). Tour leaders’ job crafting and job outcomes: the moderating role of perceived organizational support. Tour Manag Perspect.

[58] Hockey GRJ, Baddely A, Weiskrantz L (1993). Cognitive-energetical control mechanisms in the management of work demands and psychological health. Attention: selection, awareness, and control.

[59] Cheng JC, Yi O (2018). Hotel employee job crafting, burnout, and satisfaction: the moderating role of perceived organizational support. Int J Hosp Manag.

[60] Shin Y, Hur WM, Park K, Hwang H (2020). How managers’ job crafting reduces turnover intention: the mediating roles of role ambiguity and emotional exhaustion. Int J Environ Res Public Health.

[61] Demerouti E, Bakker AB, Halbesleben JR (2015). Productive and counterproductive job crafting: a daily diary study. J Occup Health Psychol.

[62] Kosenkranius MK, Rink FA, De Bloom J, Van Den Heuvel M (2020). The design and development of a hybrid off-job crafting intervention to enhance needs satisfaction, well-being and performance: a study protocol for a randomized controlled trial. BMC Public Health.

[63] Van den Heuvel M, Demerouti E, Peeters MCW, MCW JDJ, Peeters S, Sjollema HDZ (2012). Succesvol job craften door middel van een groepstraining [Succesful job crafting through group training]. Scherp in Werk: Vijf routes naar optimale inzetbaar-heid.

[64] Van Wingerden J, Bakker AB, Derks D (2017). The longitudinal impact of a job crafting intervention. Eur J Work Organ Psychol..

[65] Van Wingerden J, Derks D, Bakker AB (2017). The impact of personal resources and job crafting interventions on work engagement and performance. Hum Resour Manag.

[66] Chang JH, Huang CL, Lin YC (2015). Mindfulness, basic psychological needs fulfillment, and well-being. J Happiness Stud.

[67] Awa WL, Plaumann M, Walter U (2010). Burnout prevention: a review of intervention programs. Patient Educ Couns.

[68] Zheng Y, Zhou Z, Liu Q, Yang X, Fan C (2019). Perceived stress and life satisfaction: a multiple mediation model of self-control and rumination. J Child Fam Stud.

[69] Kim S, Park Y, Niu Q (2017). Micro-break activities at work to recover from daily work demands. J Organ Behav.

[70] von Dreden C, Binnewies C (2017). Choose your lunch companion wisely: the relationships between lunch break companionship, psychological detachment, and daily vigor. Eur Jo Work Organ Psychol.

[71] Bennett AA, Gabriel AS, Calderwood C (2020). Examining the interplay of micro-break durations and activities for employee recovery: a mixed-methods investigation. J Occup Health Psychol.

[72] Kühnel J, Zacher H, De Bloom J, Bledow R (2017). Take a break! Benefits of sleep and short breaks for daily work engagement. Eur J Work Organ Psychol..

